# Supplementary material for: Pillar[6]arene acts as a biosensor for quantitative detection of a vitamin metabolite in crude biological samples
Source: Commun Chem. 2020 Dec 7;3:183. doi: 10.1038/s42004-020-00430-w (PMC9814258; doi:10.1038/s42004-020-00430-w)
Supplement: Supplementary file 1 — Supplementary Information [file 42004_2020_430_MOESM1_ESM.docx]

Supplementary Information

**Pillar[6]arene acts as a biosensor for quantitative detection of a vitamin metabolite in crude biological samples**

Masaya Ueno^1,2,10^, Takuya Tomita^3,10^, Hiroshi Arakawa^4^, Takahiro Kakuta^2,3^, Tada-aki Yamagishi^3^, Jumpei Terakawa^5^, Takiko Daikoku^5^, Shin-ichi Horike^6^, Sha Si^1,2^, Kenta Kurayoshi^1^, Chiaki Ito^1^, Atsuko Kasahara^7^, Yuko Tadokoro^1,2^, Masahiko Kobayashi^1,2^, Tsutomu Fukuwatari^8^, Ikumi Tamai^4^, Atsushi Hirao^1,2^*, Tomoki Ogoshi^2,9^*

^1^Division of Molecular Genetics, Cancer and Stem Cell Research Program, Cancer Research Institute, Kanazawa University, Kakuma-machi, Kanazawa, Ishikawa, 920-1192, Japan

^2^WPI Nano Life Science Institute (WPI-Nano LSI), Kanazawa University, Kakuma-machi, Kanazawa, Ishikawa, 920-1192, Japan

^3^Graduate School of Natural Science and Technology, Kanazawa University, Kakuma-machi, Kanazawa, Ishikawa, 920-1192, Japan

^4^Faculty of Pharmaceutical Sciences, Institute of Medical, Pharmaceutical and Health Sciences, Kanazawa University, Kakuma-machi, Kanazawa, Ishikawa, 920-1192, Japan

^5^Institute for Experimental Animals, Advanced Science Research Center, Kanazawa University, Takara-machi, Kanazawa, 920-8641, Japan

^6^Division of Functional Genomics, Advanced Science Research Center, Kanazawa University, Takara-machi, Kanazawa, 920-8641, Japan

^7^Institute for Frontier Science Initiative, Kanazawa University, Kakuma-machi, Kanazawa, Ishikawa 920-1192, Japan

^8^Department of Nutrition, School of Human Cultures, The University of Shiga Prefecture, 2500 Hassaka, Hikone, Shiga 522-8533, Japan

^9^Department of Synthetic Chemistry and Biological Chemistry, Graduate School of Engineering Kyoto University, Kyoto, 615-8510, Japan

^10^These authors contributed equally to this work

***Correspondence:**

ahirao@staff.kanazawa-u.ac.jp (A.H.) and ogoshi@sbchem.kyoto-u.ac.jp (T.O.)

**Supplementary Note 1**

**Validation of functional Nnmt deficiency in the *Nnmt* KO mouse**

To verify that the *Nnmt* KO mouse had no mRNA that coded for functional Nnmt protein, we performed reverse transcription (RT)-PCR. Total RNA was purified from liver and transcribed into cDNA, and then cDNAs encoding Nnmt protein were amplified by PCR. PCR primers (Nnmt Fw1 and Rev1) were designed upstream and downstream of exon 2 to understand the structure of the exon boundaries. Agarose gel electrophoresis indicated that one major (#1 in wild-type (Wt), and #3 in *Nnmt* KO mouse) and one minor (#2 in Wt, and #4 in *Nnmt* KO mouse) transcript were detected (Supplementary Figure 9a). Each PCR product (from #1 to #4) was purified by gel extraction and individual DNA sequences were determined by subcloning. The sequences of the major (#1) and minor (#2) PCR products completely corresponded to the annotated mouse *Nnmt*-001 and 002 in the public database (Vertebrate Genome Annotation (VEGA) database), respectively (Supplementary Figures 9b and 10). In the *Nnmt* KO mouse, exon 1 of both cDNAs (#3 and #4) was directly connected to exon 3 without any insertion or deletion that would have caused a frameshift in the open reading frame of the Nnmt protein. We also designed other primer pairs (Nnmt Fw2 and Rev2) for amplification of the whole coding sequence of Nnmt, and performed RT-PCR. The cDNA sequences of the PCR products were directly determined without subcloning. In the electropherogram, we found that only a single peak per base was detected for almost all single nucleotides in the whole exon 2 in the Wt sequence and around the junction between exon 1 and 3 in the *Nnmt* KO sequence. These data suggests that no unexpected alternative splicing variant was transcribed in the *Nnmt* KO mouse (Supplementary Figure 11 and 12). Furthermore, while *Nnmt* is highly expressed in liver, no Nnmt protein was detected in the liver of the *Nnmt* KO mouse from the western blot analysis (Supplementary Figure 13). These data strongly demonstrate that the *Nnmt* KO mouse has no functional Nnmt protein.

**Supplementary Figures**

**
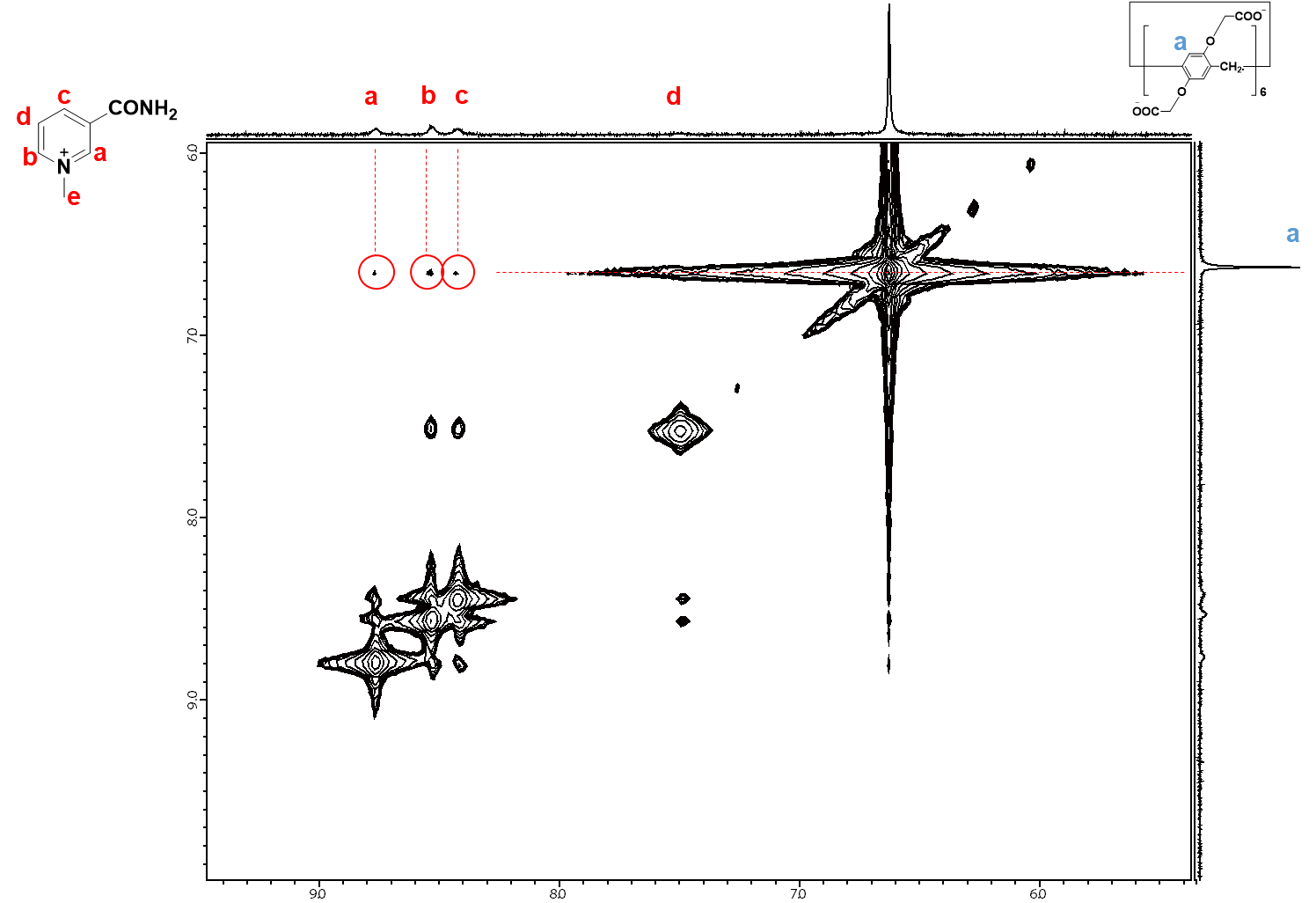
**

**Supplementary Fig. 1. 2D-NOESY study of the mixture of P6A (5 mM) and 1-MNA (5 mM) in D_2_O.**

**
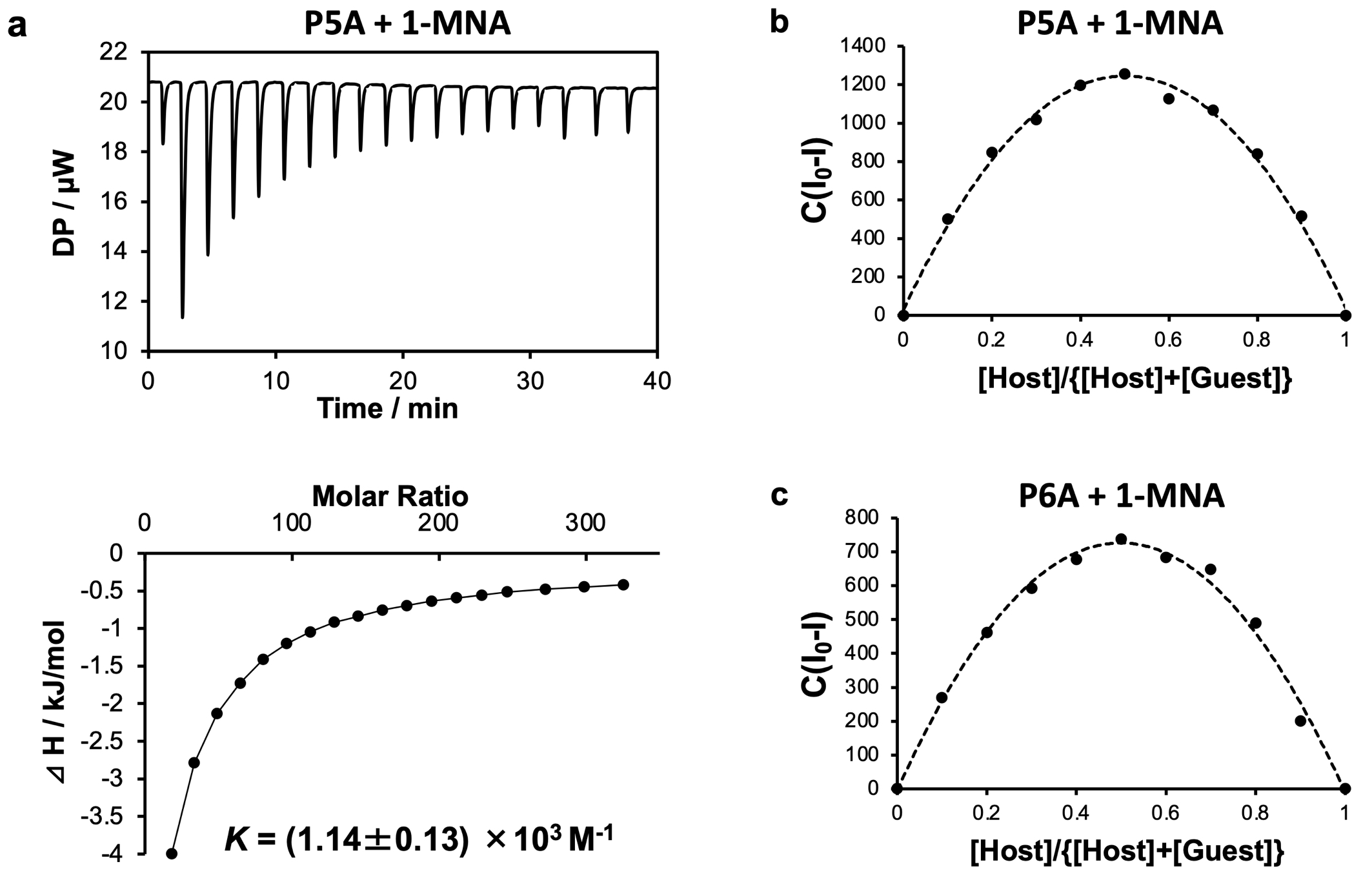
**

**Supplementary Fig. 2. ITC data for P5A and 1-MNA**

(a) Representative raw (top) and integrated heat (bottom) plots.

(b, c) Job’s plot for the interaction of 1-MNA and (b) P5A or (c) P6A.


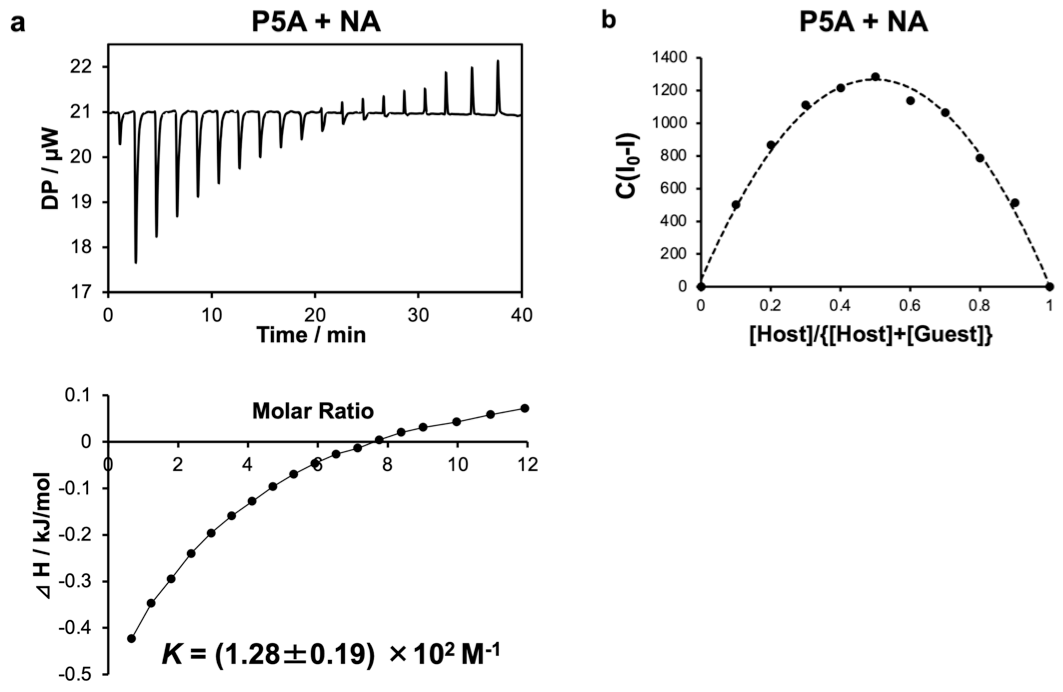


**Supplementary Fig. 3. ITC data for P5A and nicotinamide**

(a) Representative raw (top) and integrated heat (bottom) plots.

(b) Job’s plot for the interaction of P5A and nicotinamide (NA).


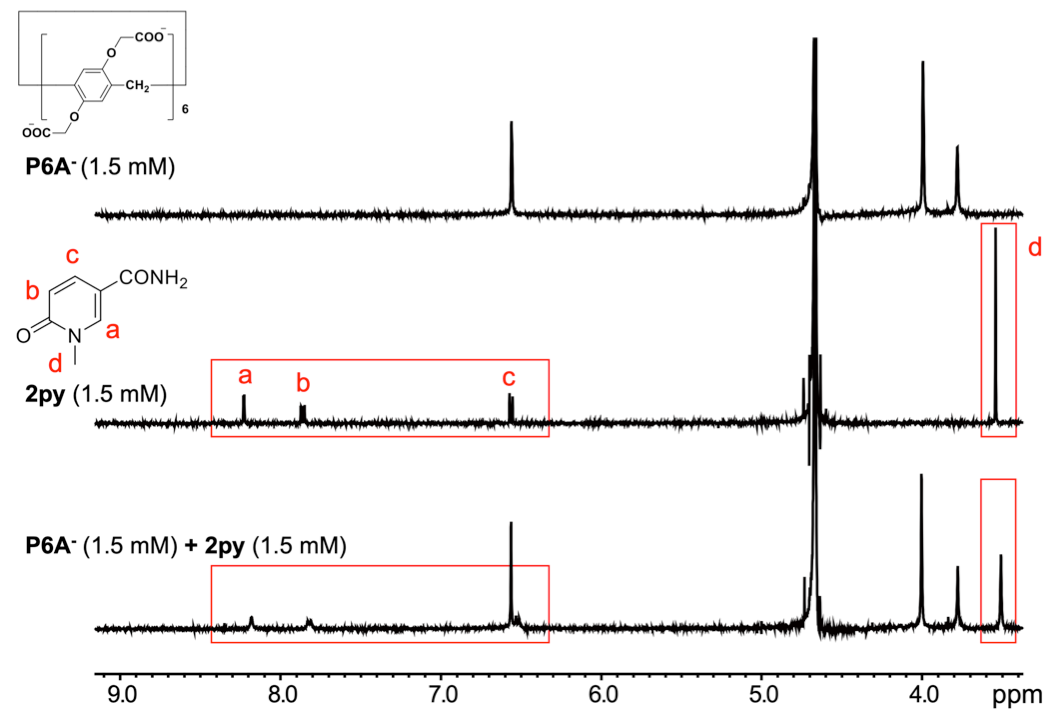


**Supplementary Fig. 4. NMR spectra for P6A and 2py and a combination of the two**

Partial ^1^H NMR spectra for P6A, 2py, and P6A and 2py. The proton peaks of 2py are indicated by red rectangles.

**
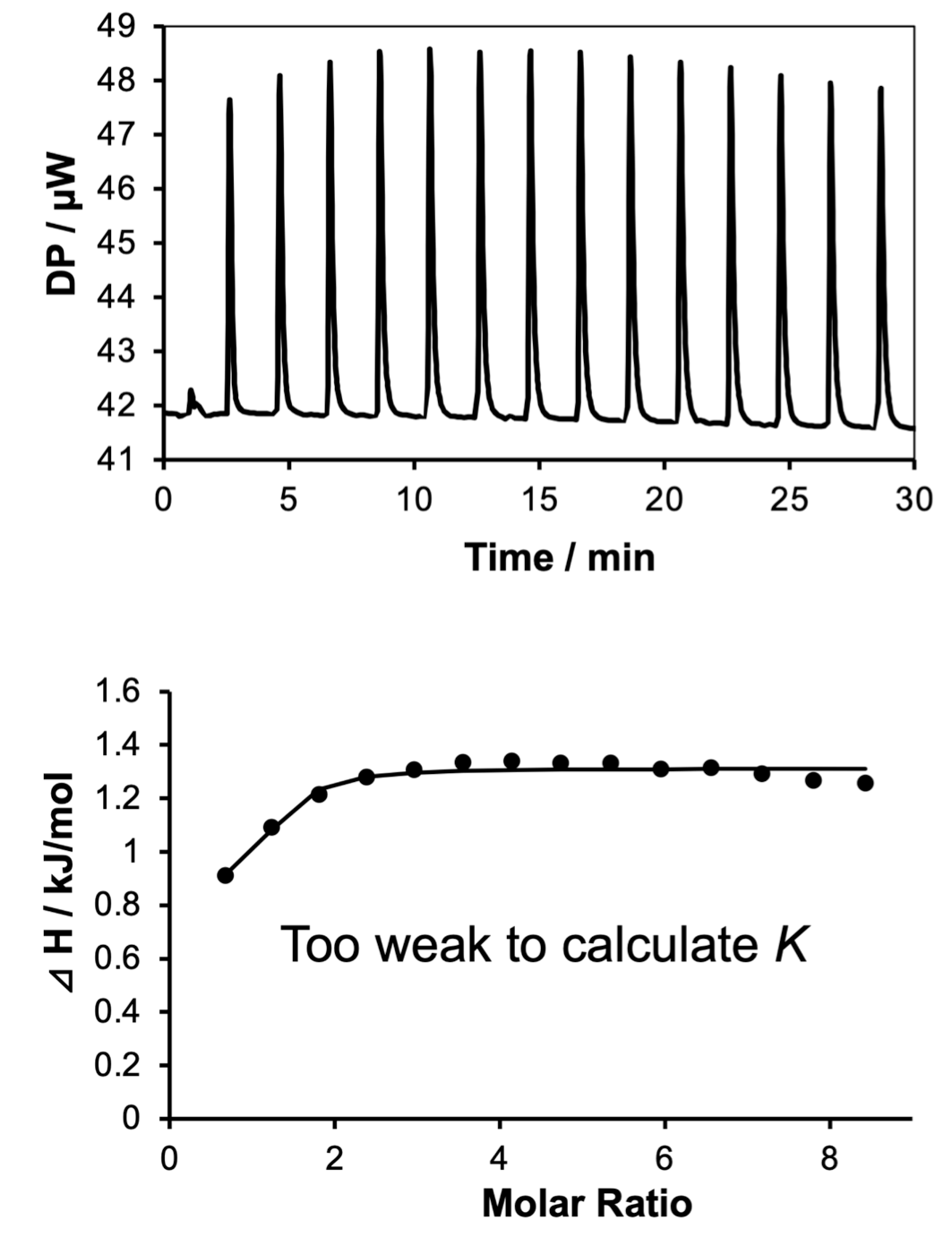
**

**Supplementary Fig. 5. ITC data for the interaction of P6A and 2py**

Representative (top) raw and (bottom) integrated heat plots for the interaction of P6A and 2py.


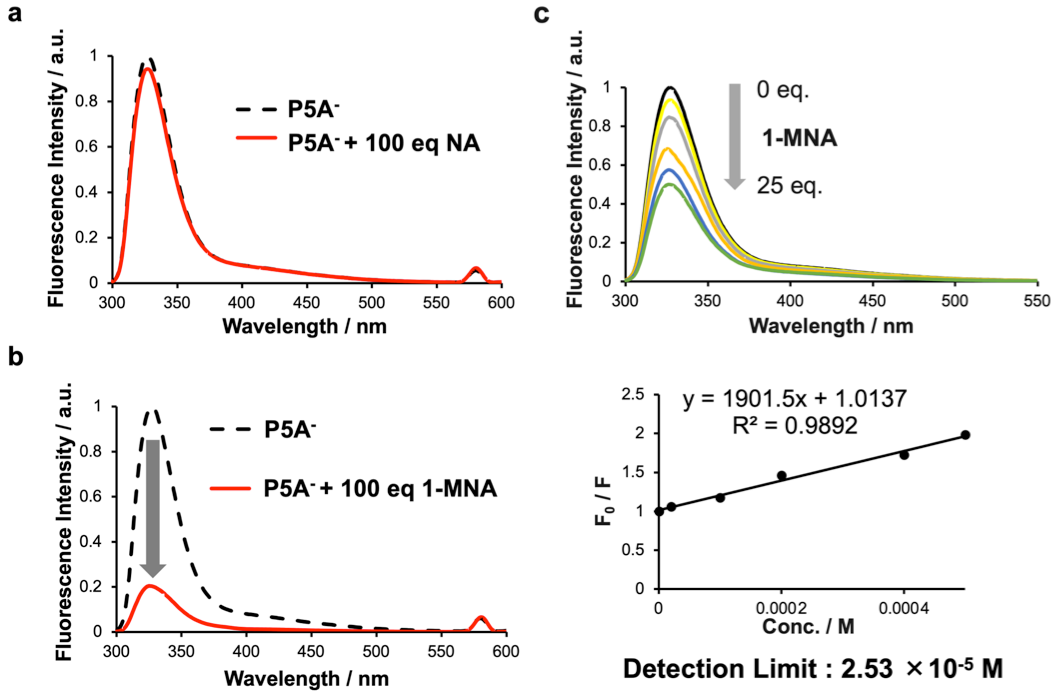


**Supplementary Fig. 6**. **Fluorescence spectra of P5A**

(a and b) Fluorescence spectra of P5A with or without 100 eq. of (a) nicotinamide or (b) 1-MNA.

(c) Fluorescence spectra of P5A with different concentrations of 1-MNA. Bottom: the linear least-squares analysis to calculate the detection limit of 1-MNA by P5A.

**
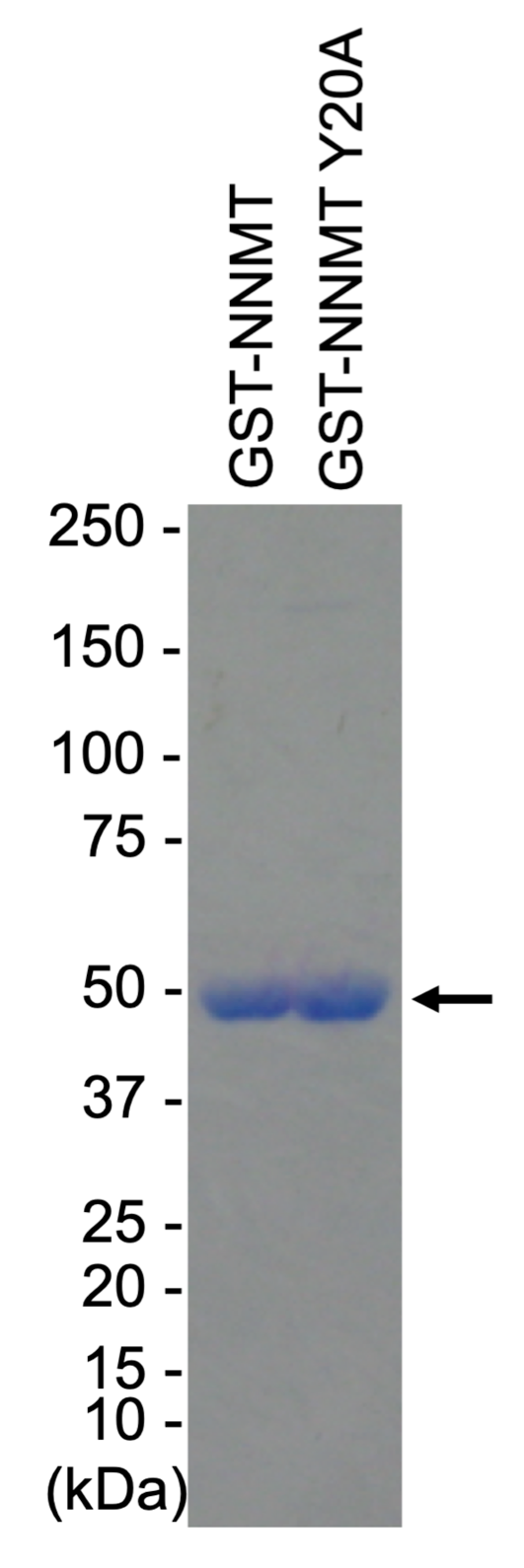
**

**Supplementary Fig. 7. Purification of recombinant GST-NNMT proteins**

SDS-PAGE analysis of purified GST-fusion proteins. Proteins purified from *E. coli* were analysed on a 4%–20% (w/v) polyacrylamide gradient gel, and visualised with Coomassie Brilliant Blue staining. Uncropped gel image is provided in Supplementary Fig. 16.

**
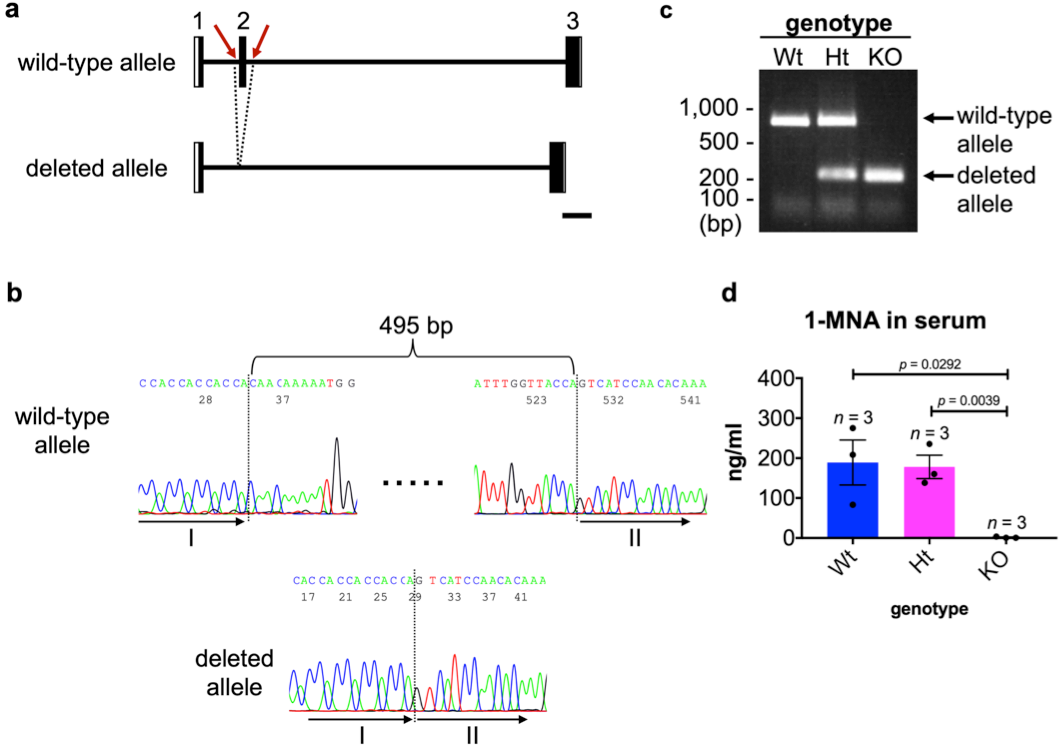
**

**Supplementary Fig. 8. Generation and characterisation of the *Nnmt* knockout mouse**

(a) Schematic representation of the genomic structure of the mouse *Nnmt* gene. Exons are boxed and numbered. Open boxes represent 5’- and 3’-UTR. Black boxes represent the protein-coding region. Arrows indicate the location of the two target sites. Scale bar, 1kbp.

(b) PCR-based genotype analysis. The 741 bp band corresponds to the endogenous wild-type *Nnmt* gene; the 246 bp band corresponds to the *Nnmt* knockout allele.

(c) Representative Sanger sequence results of the wild-type (Wt) and *Nnmt* KO allele. Deletion of a 495 bp nucleotide was observed in the *Nnmt* KO allele. Data are from one representative of three independent experiments with similar results: genomic DNA sequences from wild-type (*n* = 3) and *Nnmt* KO (*n* = 3) mice were examined.

(d) Quantification of 1-MNA in serum. 1-MNA was not detectable in serum from the *Nnmt* KO mouse. Abbreviations: Wt, wild-type; Ht, heterozygous; and KO, knockout.

Error bars represent mean ± s.e.m. Statistical significance was determined using an unpaired two-tailed Student’s t-test. Uncropped gel image is provided in Supplementary Fig. 16.


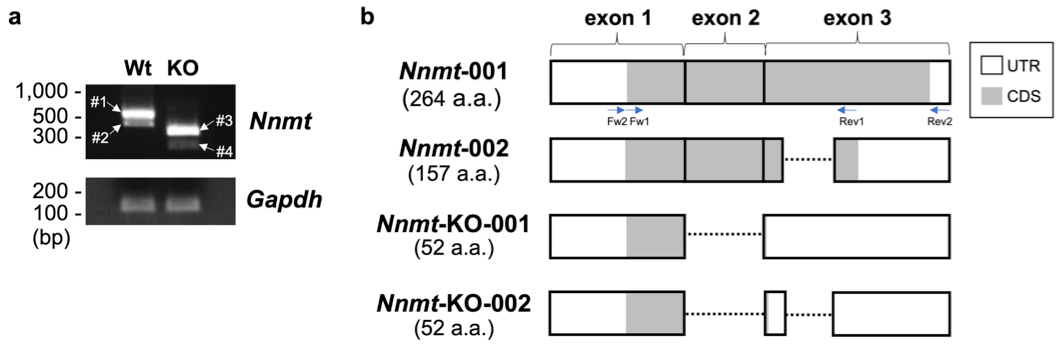


**Supplementary Fig. 9. Detection of *Nnmt* splicing variants by RT-PCR**

(a) One major and several minor RT-PCR products were observed in wild-type (Wt: #1 and #2), and *Nnmt* KO mouse (KO: #3 and #4)

(b) Schematic drawing of the splicing variants of *Nnmt* mRNA. The exon and intron structures of *Nnmt*-001 and 002 are identical to the annotated sequence in the VEGA public database. Open boxes represent the 5′- and 3′-untranslated region (UTR). Grey boxes represent the region of the protein coding sequence (CDS). Dotted lines indicate a region lacking from the full length of *Nnmt*-001. Arrows indicate each position and direction of primers used in the PCR reaction. a.a., amino acid. *Gapdh*, internal control. Uncropped gel images are provided in Supplementary Fig. 16.


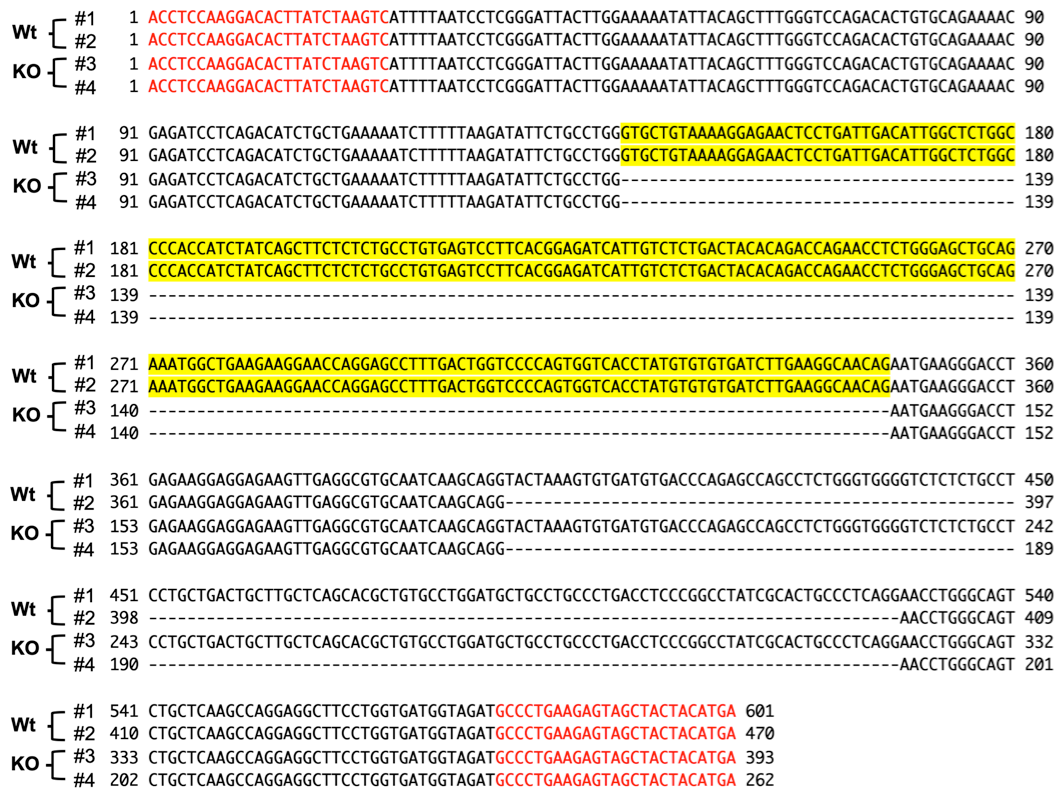


**Supplementary Fig. 10. Comparison of the cDNA sequence of each splicing variant of *Nnmt* mRNA.**

Sequences determined in this work are aligned. The sequences of exon 2 are highlighted. Red characters indicate the primer sequences.


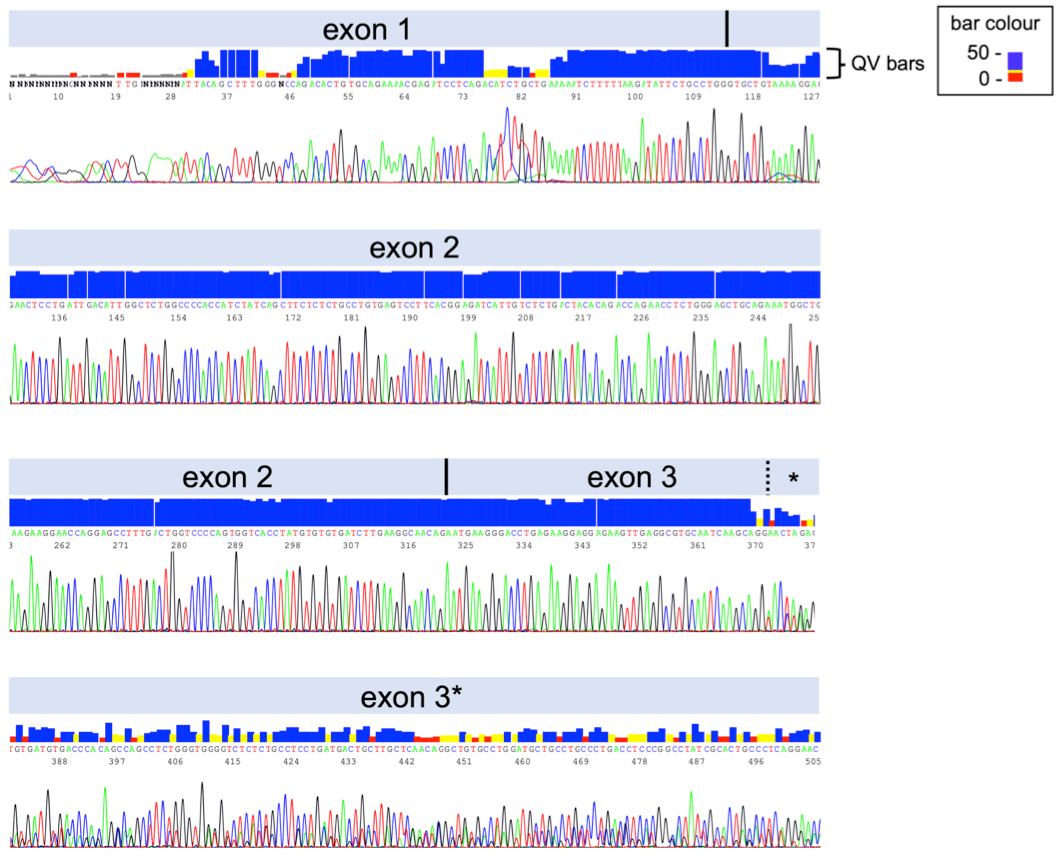


**Supplementary Fig. 11. Direct sequence of the *Nnmt* cDNA derived from a wild-type mouse.**

The electropherogram is shown. The quality value (QV) indicates an estimation of the quality of the identification of bases generated by the sequencing. The vertical bars indicate the boundaries between two exons. The dotted vertical bars indicate the boundary of exon 3, whose sequence is lacking in the *Nnmt*-002 cDNA. The region indicated with an asterix* contains a mixed sequence of *Nnmt*-001 and -002 cDNA.


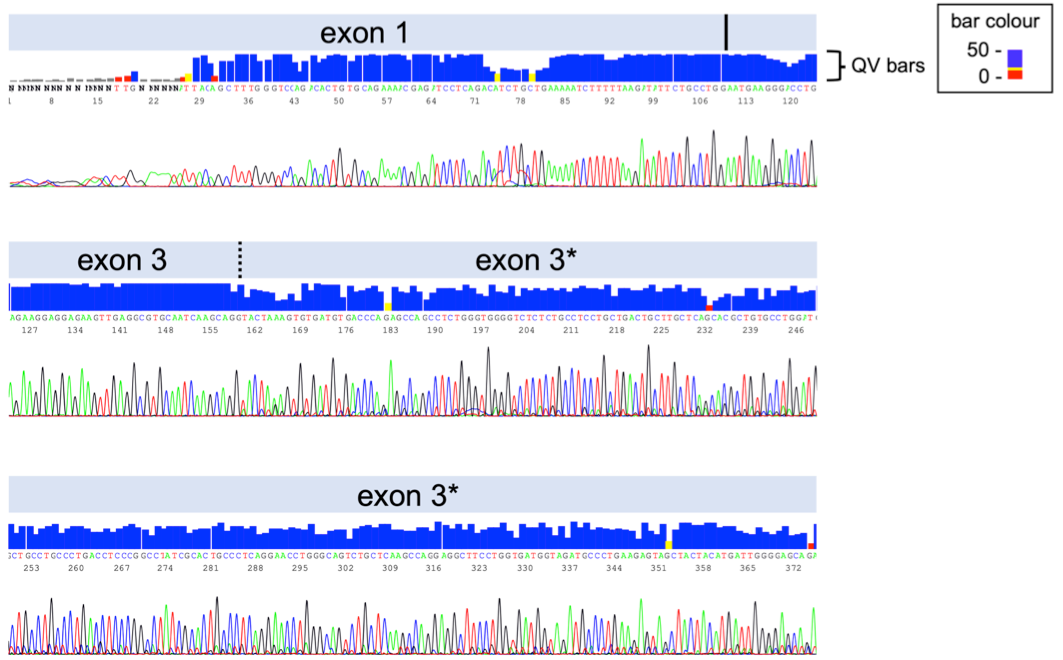


**Supplementary Fig. 12. Direct sequence of the *Nnmt* cDNA derived from the *Nnmt* KO mouse.**

The electropherogram is shown. QV, Quality Value. The vertical bars indicate the boundaries between two exons. The dotted vertical bars indicate the boundary of exon 3, whose sequence is lacking in the *Nnmt*-KO-002 cDNA. The region indicated with an asterix* contains a mixed sequence of *Nnmt*-KO-001 and -KO-002 cDNA.

**
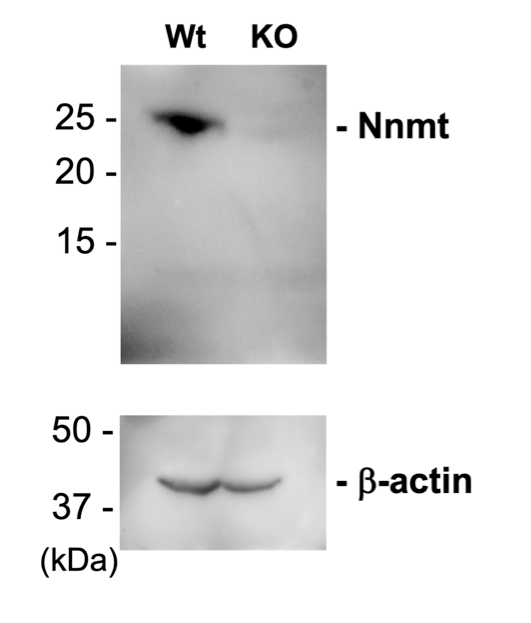
**

**Supplementary Fig. 13. Western blot analysis of wild-type and *Nnmt* KO livers.**

Presence of a band at ~25 kDa in the liver from wild-type (Wt) mouse but not *Nnmt* KO mouse (KO) is seen with the anti-Nnmt antibody. β-Actin labelling was used as a loading control. Uncropped blot images are provided in Supplementary Fig. 16.


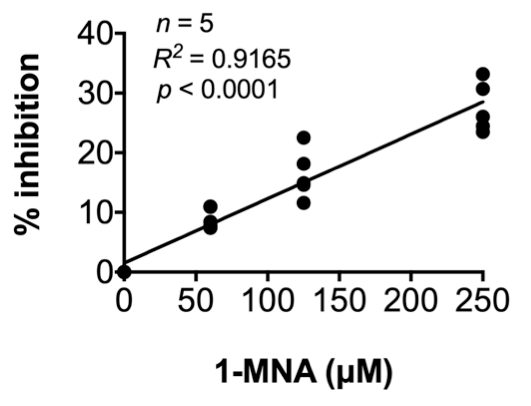


**Supplementary Fig. 14. Validation of the specificity of P6A to 1-MNA by examination of dilution of 1-MNA into *Nnmt* KO-derived urine.**

1-MNA was diluted in *Nnmt* KO mouse-derived urine, and fluorescence measurements were performed. The line represents the linear regression calculation (*n* = 5), and the doses of 1-MNA tested were 0, 60, 125, and 250 µM.


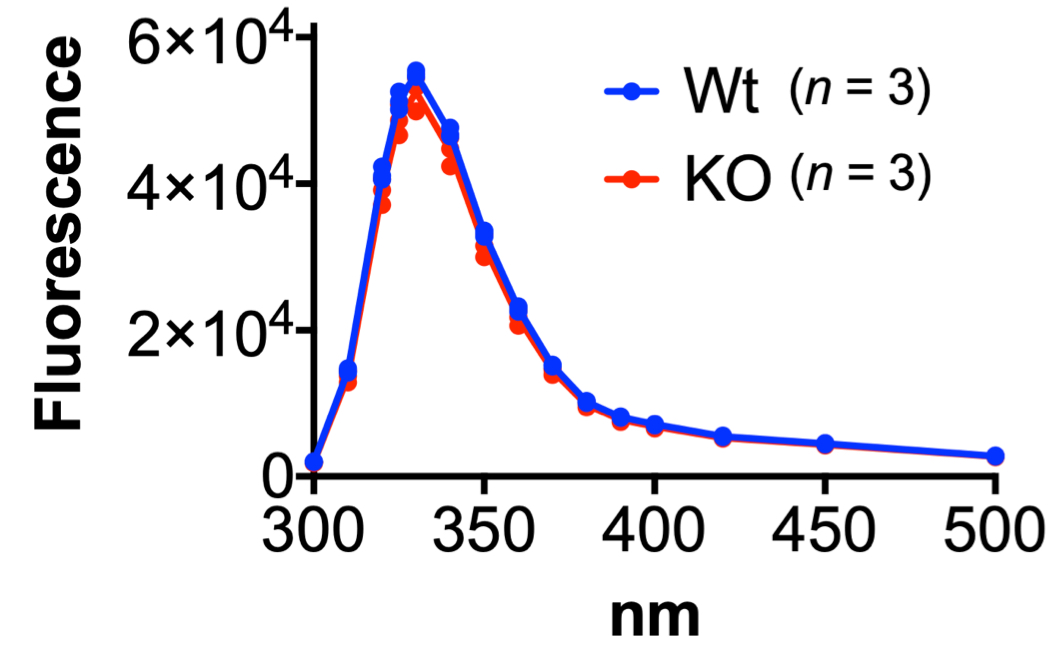


**Supplementary Fig. 15. P6A is not useful to detect 1-MNA in blood samples.**

Fluorescence spectra of P6A for detection of 1-MNA in plasma. No significant difference was observed between the wild-type (wt) and *Nnmt* KO (KO) mouse.


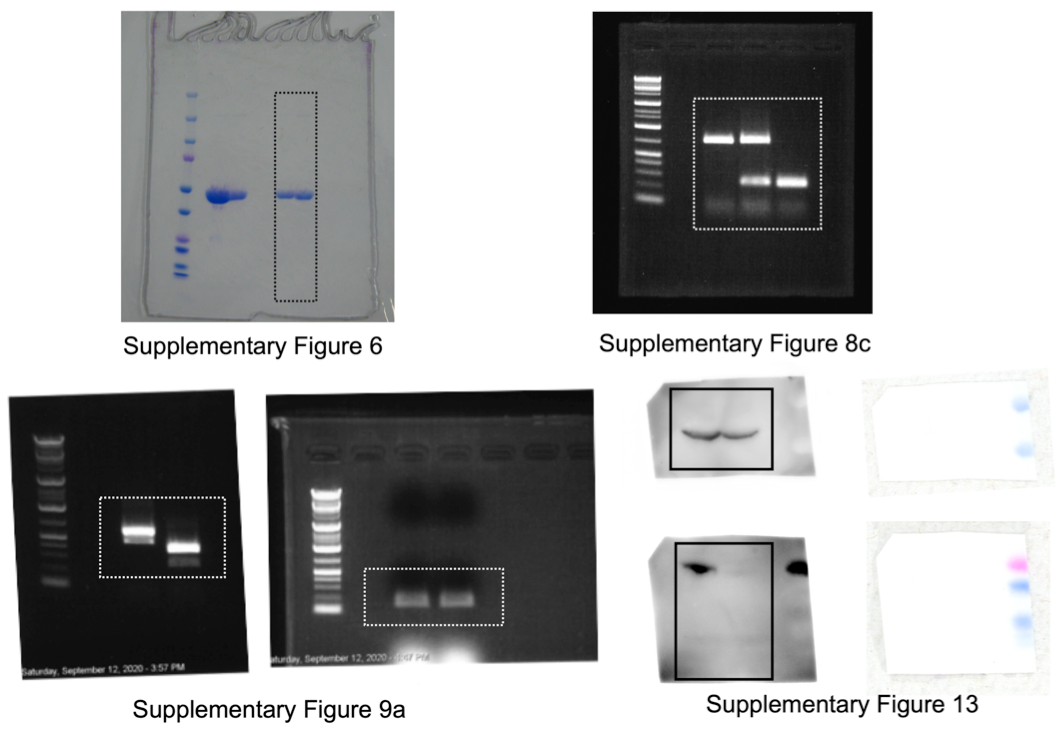


**Supplementary Fig. 16. Uncropped images of gels and blots for Supplementary Figure 6, 8c, 9a, and 13.**
